# Infected foot ulcers in male and female diabetic patients: a clinico-bioinformative study

**DOI:** 10.1186/1476-0711-9-2

**Published:** 2010-01-14

**Authors:** Shazi Shakil, Asad U Khan

**Affiliations:** 1 Interdisciplinary Biotechnology Unit, Aligarh Muslim University, Aligarh, India-202002

## Abstract

**Background:**

The study aimed at (i) characterizing the mode of transmission of *bla*_CTX-M _and *bla*_TEM-1 _among extended-spectrum-β-lactamase (ESBL)-producing *Escherichia coli *strains isolated from infected diabetic foot ulcers, and (ii) identifying the risk factors for "sex-associated multidrug resistant Gram-negative bacterial (MDRGNB)-infection status" of the ulcers.

**Methods:**

Seventy-seven diabetic patients having clinically infected foot ulcers were studied in a consecutive series. The *E. coli *strains isolated from the ulcers were screened for *bla*_CTX-M_, *bla*_TEM-1_, *armA*, *rmtA *and *rmtB *during the 2-year study-period. PCR amplified *bla*_CTX-M _genes were cloned and sequenced. Enterobacterial repetitive intergenic consensus (ERIC)-PCR was used for the analysis of genetic relatedness of the ESBL-producers. Risk factors for "sex-associated MDRGNB-infection status" of ulcers were assessed. Modeling was performed using Swiss-Model-Server and verified by Procheck and verify3D programmes. Discovery Studio2.0 (Accelrys) was used to prepare Ramachandran plots. Z-scores were calculated using 'WHAT IF'-package. Docking of cefotaxime with modeled CTX-M-15 enzyme was performed using Hex5.1.

**Results:**

Among 51 *E. coli *isolates, 14 (27.5%) ESBL-producers were identified. Only 7 Class1 integrons, 2 *bla*_CTX-M-15_, and 1 *bla*_TEM-1 _were detected. Ceftazidime and cefotaxime resistance markers were present on the plasmidic DNA of both the *bla*_CTX-M-15 _positive strains and were transmissible through conjugation. The residues Asn132, Glu166, Pro167, Val172, Lys234 and Thr235 of CTX-M-15 were found to make important contacts with cefotaxime in the docked-complex. Multivariate analysis proved 'Glycemic control at discharge' as the single independent risk factor.

**Conclusions:**

Male diabetic patients with MDRGNB-infected foot ulcers have poor glycemic control and hence they might have higher mortality rates compared to their female counterparts. Plasmid-mediated conjugal transfer, albeit at a low frequency might be the possible mechanism of transfer of *bla*_CTX-M-15 _resistance marker in the present setting. Since the docking results proved that the amino acid residues Asn132, Glu166, Pro167, Val172, Lys234 and Thr235 of CTX-M-15 (enzyme) make important contacts with cefotaxime (drug) in the 'enzyme-drug complex', researchers are expected to duly utilize this information for designing more potent and versatile CTX-M-inhibitors.

## Background

Multidrug resistant Gram-negative bacteria (MDRGNB) are a major therapeutic challenge both in hospital and community settings [1]. We have recently reported a high prevalence of extended-spectrum beta lactamase (ESBL)-producing bacteria in the neonatal intensive care unit of Aligarh hospital, India [2]. ESBLs are often plasmid-associated and there can be cross-species dissemination of these plasmids. Moreover, these plasmids often carry genes for co-resistance to other antibiotics such as aminoglycosides, fluoroquinolones, tetracyclines, chloramphenicol and sulfamethoxazole-trimethoprim. Concomitant β-lactam and aminoglycoside resistance involving *armA *and *rmtB *genes is increasingly being reported [3].

Foot ulcers are a very common complication of type 1 and type 2 diabetes. The Indian diabetic population is expected to increase to 57 million by 2025 [4]. Individuals with diabetes have at least a 10-fold greater risk of being hospitalized for soft tissue and bone infections of the foot than the individuals without diabetes [5]. Infections of foot ulcers by ESBL-producing MDRGNB in diabetic patients have been described frequently [6]. Due to non-use of standard microbiological techniques, there is a paucity of ESBL data concerning diabetic foot ulcers particularly in India.

CTX-Ms have become the most prevalent ESBLs worldwide. It is of due importance to know the amino acid residues crucial to the interaction between cefotaxime and CTX-M-15, as this enzyme is increasingly being reported from this part of the world together with TEM-1 [7]. Moreover, *Escherichea coli *is one of the most common infecting organisms isolated from soft tissue infections [8]. Accordingly we looked for the mode of transmission of *bla*_CTX-M _and *bla*_TEM-1 _resistance markers among the *E. coli *strains isolated from infected foot ulcers of diabetic patients admitted to the endocrinology ward of the Aligarh hospital. The study aimed at (i) characterizing the mode of transmission of *bla*_CTX-M _and *bla*_TEM-1 _among ESBL-producing *E. coli *strains isolated from infected diabetic foot ulcers, and (ii) identifying the risk factors for "sex-associated MDRGNB infection status" of the ulcers.

## Methods

### Collection of bacterial strains and patients' details

The study was conducted at Aligarh hospital, India (from April 2007 to November 2008). Seventy-seven diabetic patients having clinically infected foot ulcers admitted to the endocrinology ward were studied in a consecutive series. Wagner classification was employed to grade the ulcers [9]. Thirty-two study factors were recorded for each patient. BMI i.e. Body Mass Index (< 18.5 kg/m^2 ^= underweight; 18.5-22.9 kg/m^2 ^= normal weight; 23.0- 24.9 kg/m^2 ^= overweight), presence of nephropathy (creatinine ≥ 150 μmol/l or presence of micro- or macroalbuminuria), neuropathy (absence of perception of the Semmes-Weinstein monofilament at 2 of 10 standardized plantar sites on either foot) and peripheral vascular disease (ischemic symptoms and intermittent claudication or rest pain, with or without absence of pedal pulses), were some of them. Osteomyelitis was diagnosed on suggestive changes in the radiographs and bone scans. Regarding a fasting blood glucose level of < 110 mg/dl and/or postprandial level of < 160 mg/dl as glycemic control, the number of patients achieving glycemic control over the hospital stay was compared with respect to sex. In case a patient had multiple ulcers, specimens from all the ulcers were taken. If any of the ulcers was found to be infected with MDRGNB, the patient was grouped in the MDRGNB-infected category. Fasting HDL and VLDL were measured and LDL was calculated by subtracting their sum from total cholesterol. Clinical assessment for signs of infection (swelling, exudate, surrounding cellulitis, odor, tissue necrosis, crepitation, and pyrexia) was made. Ulcer size was determined by multiplying the longest and widest diameters and expressed in centimeters squared. Demographic and other relevant details were retrieved from medical records.

### Microbiological methods

Culture specimens were obtained at the time of admission, after the surface of the wound had been washed vigorously by saline, and followed by debridement of superficial exudates. Specimens were obtained by scraping the ulcer base or the deep portion of the wound edge with a sterile curette. The soft tissue specimens were quickly sent to the laboratory and processed for Gram-negative bacteria. Standard methods for isolation and identification of bacteria were used [10]. Antimicrobial susceptibility testing of the isolates was performed by the standard disc diffusion method as recommended by the Clinical and Laboratory Standards Institute (CLSI) [11]. The test-antibiotics included gentamycin, tobramycin, amikacin, kanamycin, streptomycin, cefalothin, cefazolin, cefuroxime, cefoxitin, ceftazidime, cefotaxime, ceftriaxone, cefepime, ciprofloxacin, norfloxacin, tetracycline, piperacillin, imipenem as well as combinations such as amoxyclav and piperacillin/tazobactam. Minimum Inhibitory Concentrations (MICs) for the same antimicrobials were determined by the CLSI microbroth dilution method [11]. The ESBL synergy test for the study isolates was performed as described by Jarlier et al [12]. For this study, bacteria resistant to at least 4 of the test antibiotics were designated as MDRGNB. All ESBL-producers were considered as MDRGNB.

### Molecular typing and PCR

Enterobacterial repetitive intergenic consensus (ERIC)-PCR was used for the analysis of genetic relatedness of the ESBL-producers as described elsewhere [13]. A search for *bla*_CTX-M_, *bla*_TEM-1_, *armA*, *rmtA *and *rmtB *genes in the genomic and plasmidic DNA of isolates which were positive for ESBL synergy test was performed by PCR amplification, as described previously [14-16]. Plasmids were obtained by the method of Kado and Liu [17]. Screening of integrons was also performed as described elsewhere [18].

### Cloning and sequencing

PCR amplified *bla*_CTX-M _genes, were ligated into 'pDrive-easy-PCR-cloning-vector' and transformed into competent *E. coli *C600 cells using Qiagen Cloningplus Kit (Qiagen, USA). Transformed colonies were selected on Luria-Bertani agar plates, containing isopropyl-β-D-thiogalactopyranoside (1 mM), 5-bromo-4-chloro-3-indolyl-β-D-thiogalactopyranoside (40 mg/L) and kanamycin (30 mg/L). Plasmid extracted from the culture of a well-isolated white colony was used as template for PCR amplifications of *bla*_CTX-M_. The same plasmids were transformed [19] into azide-resistant *E. coli *J53 cells. The transformants were selected on Luria-Bertani agar plates containing azide (250 mg/L), cefotaxime (2 mg/L) and kanamycin (30 mg/L). The transformed cells were re-checked for ESBL-production. DNA sequences were analyzed with ABI 3130 Genetic Analyzer (Applied Biosystems). The Basic Local Alignment Search Tool (BLAST) program of the National Center for Biotechnology Information http://www.ncbi.nlm.nih.gov was used to search databases for similar nucleotide sequences.

### Marker transfer experiments

*E. coli *strains that were PCR-positive for any of the tested genes were used as donors in transconjugation experiments as described elsewhere [20]. Azide-resistant *E. coli *J53 was used as the recipient. Transconjugants were selected on Luria-Bertani agar plates containing azide (250 mg/L) and either cefotaxime (2 mg/L) or gentamicin (50 mg/L).

### Statistical analysis

Qualitative variables were expressed as percentages and quantitative variables were expressed as means ± SD (Standard Deviation). Significance of the study variables for "sex-associated MDRGNB infection status" of the foot ulcers was tested by using Student's *t *test or Fisher's exact test as appropriate. All risk factors that proved to be statistically significant on univariate analysis were entered by blockwise entry in a logistic regression model after exclusion of multicollinearity and interaction between variables. A two-tailed p value of < 0.05 was taken as significant. Statistical analysis was performed using SPSS (version 11.5, Chicago). The results were again confirmed using the SISA (Simple Interactive Statistical Analysis) program http://www.quantitativeskills.com/sisa/distributions/binomial.htm

### Homology Modeling and Docking

BLAST-P was performed to retrieve suitable templates for homology-modeling using the *bla*_CTX-M _sequences obtained in this study. Protein Data Bank (PDB) IDs of these templates are shown in table 1. Models were prepared using Swiss-Model-Server [21] and verified by Procheck [22] and verify3D programmes [23]. Discovery Studio2.0 (Accelrys) was used to prepare Ramachandran plots. Z-scores were calculated using 'WHAT IF'-package. Cefotaxime was docked into each of the modeled structures employing Hex 5.1.

**Table 1:** Analysis of *bla*_CTX-M-15 _markers using bioinformatic approach and clinical profile

CTX-M-15 positive *E. coli *strain	D253	D281
ERIC profile	M1	M1

Class1 integron	+	+

*int1*	+	+

*sul1*	+	+

Sampling date	8/4/2008	14/6/2008

Sex of the patient	Female	Male

Accession no. of reference strain used for alignments	FJ668785	FJ668753

^#^PDB ID of the template retrieved for modeling	1iysA	1iysA

Cefotaxime MIC of the strain (mg/L)	256	64

Ceftazidime MIC of the strain (mg/L)	32	32

*E-total (Kj/mol)	-221.51	-168.17

*E-shape (Kj/mol)	-208	-147.5

*E-force (Kj/mol)	-13.51	-20.67

Genbank accession numbers (This study)	FJ997866	GQ145220

^#^PDB = Protein Data Bank

*E = Binding energy with cefotaxime

## Results

### General results

**Figure 1:**
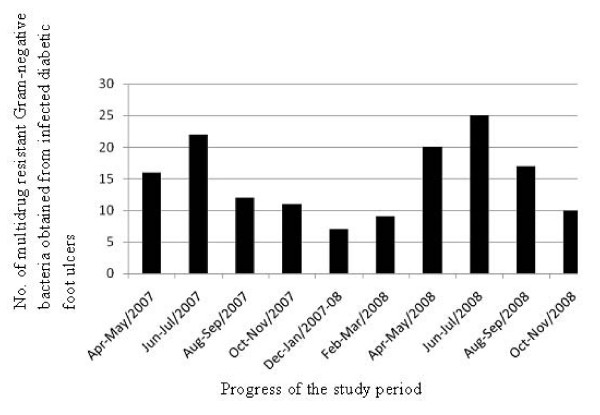
**Distribution of multidrug resistant Gram-negative bacteria obtained from infected diabetic foot ulcers divided into 2-month periods from April 2007 to November 2008**.

A total of 185 Gram-negative bacteria were isolated from 95 foot ulcer samples of the 77 diabetic patients admitted during the study-period. Out of these bacteria, 149 (80.5%) were found to be MDRGNB. Sixty-six samples which belonged to 55 patients were found to harbor these MDRGNB. Fig 1 shows the distribution of MDRGNB obtained from infected diabetic foot ulcers divided into 2-month periods from April 2007 to November 2008. Characteristics of male and female patients having MDRGNB-infected foot ulcers were recorded. Among the 55 MDRGNB-infected patients, 46 (83.6%) were males while 9 (16.4%) were females. Grade-5 ulcer was found in 1 male patient only. The majority of subjects (89.1%) had type 2 diabetes. Fifty-one (92.7%) were hypertensive, 22 (40%) had retinopathy, 37 (67.3%) had nephropathy, 34 (61.8%) had neuropathy and 33 (60%) had peripheral vascular disease. Nine (16.4%) patients had osteomyelitis. Three male patients died during the hospital stay. However, there were only 2 subjects who possessed foot ulcer infected with more than 1 ESBL-producing strain.

### Antimicrobial susceptibility and ESBL-production

Forty-three (28.9%) ESBL-producers were identified among the 149 MDRGNB. The highest ESBL production was noted in *Pseudomonas aeruginosa *(20/61; 32.8%), followed by *E. coli *(14/51; 27.5%) and *K. pneumoniae *(9/38; 23.7%). All the MDRGNB showed 100% susceptibility to imipenem (data not shown). All the ESBL-producers, with the exception of *P. aeruginosa *strains were found to be susceptible to cefoxitin, imipenem and piperacillin/tazobactam while the same were resistant to ceftazidime, cefotaxime and piperacillin. However, the ESBL producing strains of *P. aeruginosa *were invariably resistant to cefoxitin. Amikacin was the most effective of all the aminoglycosides against ESBL-producers. Imipenem alone and the piperacillin/tazobactam combination were found to be the therapies of choice. Hence, the 43 ESBL-producers could be broadly divided into 3 different resistance patterns. Strains resistant to aminoglycosides and quinolones but susceptible to cefepime constituted pattern 1. Pattern 2 comprised of strains resistant to cefepime but susceptible to either aminoglycosides or quinolones or both. Strains resistant to all the three mentioned classes of antibiotics constituted pattern 3. Resistance pattern 1 was found to be the most common among ESBL-producers. The other aerobic Gram-negative organisms isolated from foot ulcers included *Acinetobacter baumannii *(5 strains), *Proteus *species (20 strains), *Enterobacter *species (1 strain) and *Morganella morganii *(1 strain). None of these were ESBL-producers. Only 13 anaerobes were isolated of which 8 were Gram-negative. The Gram-negative anaerobes consisted of *Veillonella *species (3 strains) and *Bacteroides *species (5 strains) none of which happened to be MDRGNB.

### PCR amplifications, cloning and sequencing of bla_CTX-M-15_

Only 2 strains were confirmed to harbor *bla*_CTX-M _by PCR. Strain D253 was positive for both *bla*_CTX-M _and *bla*_TEM-1 _while strain D281 possessed only *bla*_CTX-M_. None of the strains were positive for any of the 16S rRNA methyl transferase genes (i.e. *armA*, *rmtA *or *rmtB*). Seven integrons were detected whose sizes ranged from 600 bp to 1.5 Kb. All these integrons were found to be Class1 type and were positive for *int1 *and *sul1 *genes of the expected sizes 845 bp and 840 bp respectively. None of the genes were located in the integrons. *E. coli *C600 cells harboring cloned *bla*_CTX-M _(C600 *^bla^*^CTX-M^) were again found to be PCR-positive for the said gene in all instances. Moreover, cultures derived from C600 *^bla^*^CTX-M ^cells gave a positive ESBL synergy test. Azide resistant *E. coli *J53 cells transformed with the plasmidic DNA extracted from C600 *^bla^*^CTX-M ^cells became cefotaxime resistant. Also, cultures derived from these transformed *E. coli *J53 cells were found to be positive for the ESBL test. Sequencing results confirmed all the *bla*_CTX-M _genes obtained in this study as *bla*_CTX-M-15_.

### Plasmid mediated marker transfer and molecular typing

**Figure 2:**
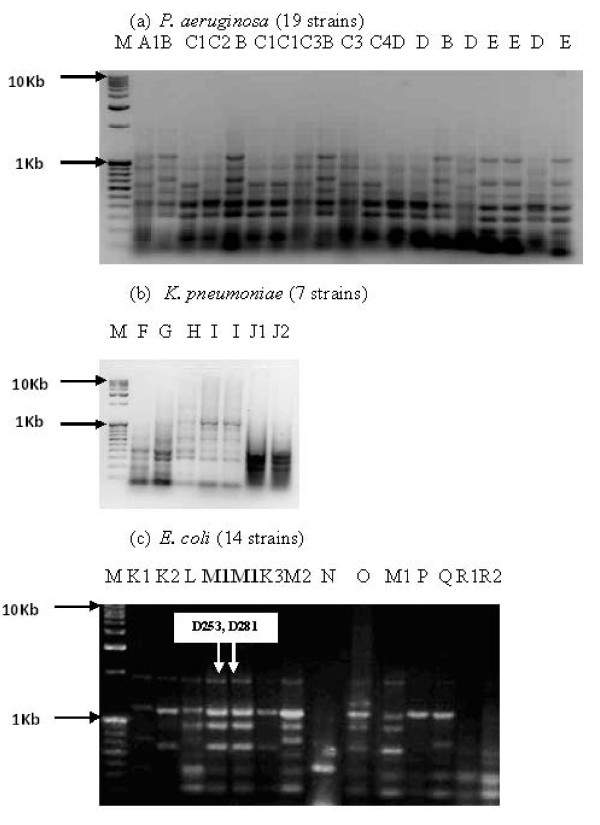
**ERIC-profiles of extended-spectrum β-lactamase producing bacteria isolated from infected foot ulcers of diabetic patients**. 'M' stands for marker (the first lane of each figure).

The ceftazidime and cefotaxime resistance markers were located on the plasmidic DNA of both the *bla*_CTX-M-15 _positive strains and were transmissible through conjugation. Quinolone (ciprofloxacin) resistance was co-transferred with cefotaxime resistance, in only one of the *bla*_CTX-M-15 _positive isolates. Transfer frequencies (Total no. of transconjugants per plate/Total no. of recepients per plate) observed were of the order 10^-5 ^(Table 2). Randomly taking a single representative strain in case of identical susceptibility patterns, ERIC-PCR was performed for 40 ESBL-producers. *P. aeruginosa, K. pneumoniae *and *E. coli *displayed 5, 5 and 8 major ERIC-profiles respectively (Fig 2). Both the *bla*_CTX-M-15 _positive strains were found to be clonally related and displayed ERIC-profile M1 (Table 1).

**Table 2:** Transconjugation frequencies, resistance markers transferred to recipient strains and MICs of antibiotics used against *bla*_CTX-M-15 _positive strains of *E. coli*

Name of the strain	D253	D281
**MIC (mg/L)**	0	0
Gentamicin (G)	128	64

Tobramycin (Tb)	64	32

Kanamycin (Ka)	64	128

Amikacin (Ak)	32	32

Streptomycin (St)	64	64

Cefazolin (Cz)	>256	>256

Ceftazidime (Ca)	32	32

Cefotaxime (Ce)	256	64

Ceftriaxone (Ci)	256	512

Cefepime (Cpm)	64	64

Ciprofloxacin (Cf)	64	64

Norfloxacin (Nx)	64	32

Piperacillin (Pc)	>256	512

Amoxyclav (Ac)	4	2

Tetracycline (T)	256	512

**Resistance pattern type**	3	3

**Resistance markers present in donor or test isolates**	G, Tb, Ka, St, Ch, Cz, Cu, Ca, T, Ce, Ci, Cpm, Cf, Nx, Pc	G, Tb, Ka, St, Ch, Cz, Cu, Ca, T, Ce, Ci, Cpm, Cf, Nx, Pc

**Resistance markers transferred to the recipient (*E. coli*J53)**	Ca, Ce, Ci, T	Ca, Ce, Ci, Cf

**Total no. of transconjugants per plate**	1.7 × 10^2^	2.1 × 10^2^

**Total no. of recepients (*E. coli*J53) per plate**	10^7^	10^7^

**Tansfer Frequency (Total No. of transconjugants per plate/Total no. of recepients per plate)**	1.7 × 10^-5^	2.1 × 10^-5^

### Homology modeling and docking

**Figure 3:**
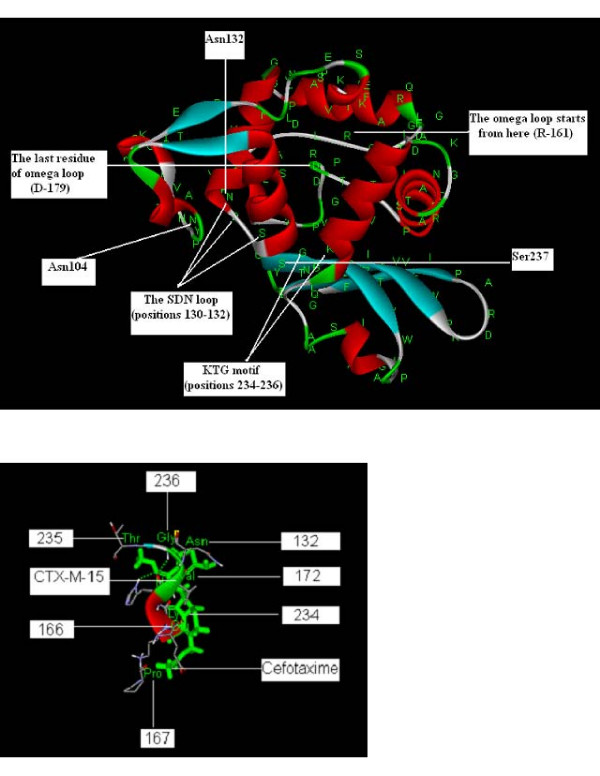
**(a) CTX-M-15 enzyme modeled from the *bla*_CTX-M-15 _gene sequence [Genbank: **FJ997866] **of *E. coli *strain D253**. The active site Ω-loop, the SDN loop and the KTG motif are labeled. A = 91.5%, B = 7.8%, C = 0.7%, D = 0.0%, where 'A', 'B', 'C' and 'D' stand for percent amino acid residues in most favored, additional allowed, generously allowed and disallowed regions of the Ramachandran plot respectively. The z-score for the modeled structure was equal to 0.027. **(b) **Amino acid residues crucial to the interaction between cefotaxime and CTX-M-15.

Over 90% of the amino acid residues in the protein structures modeled from *bla*_CTX-M-15 _genes obtained in this study were found to be present in the most favorable regions as revealed by their respective Ramachandran plots. The Ramachandran Z-score for modeled enzyme from strain D253 was found to be 0.027 (Fig 3a), which further confirms the accuracy of the modeled structure. Cefotaxime was docked into the modeled enzyme-structures. It was found that the strain (D253) having a higher cefotaxime MIC displayed a higher negative binding energy as well (Table 1). Analysis of the docked structures by Discovery Studio2.0 revealed that the amino acid residues Asn132, Glu166, Pro167, Val172, Lys234 and Thr235 make important contacts with cefotaxime (Fig 3b).

### Risk factor analysis

In the univariate analysis, many study variables including mean age, mean ulcer duration, smoking, Wagner grade and 'glycemic control at discharge' (all p = 0.00) were found to be significant for "sex-associated MDRGNB infection status" of the foot ulcers. Hence, we went for multivariate analysis. The multiple logistic regression model confirmed 'glycemic control at discharge' to be the single independent risk factor for the same. (p = 0.001). ORs and 95% CIs can be seen in Additional file 1. Hence our main finding is that, male diabetic patients having MDRGNB-infected foot ulcers have poor glycemic control during the hospital stay.

### Nucleotide sequence accession numbers

The accession numbers of the *E. coli *genes sequenced in this study are [GenBank: FJ997866], [GenBank: FJ997867] and [GenBank: GQ145220].

## Discussion

This is the first study to report on risk factors for "sex-associated MDRGNB-infection status" of diabetic foot ulcers from this region of India (Uttar Pradesh). We do not claim that these 77 patients represent the entire region. Hence, similar studies from this region and across the globe that could help in updating the empirical antibiotic regimen in diabetic foot ulcers are highly recommended. We regret that we could not use bone biopsy sample in case of osteomyelitis. Bone biopsy may be traumatic to patient and is not routinely performed in our hospital. Moreover, Pellizzer et al. [24] have reported that samples taken from the base of the wound after debridgement are adequate to identify the infecting organism, which we needed. Infections due to multidrug resistant organisms in hospitalized patients with diabetic foot ulcers have been reported previously [25]. Compared with earlier reports, we recovered fewer anaerobic species [26]. Our patients did not have chronic draining wounds, and very few had gangrene associated with their infections. This may be an indication of fewer anaerobic species among nonthreatening lower-extremity infections, which has been reported earlier [27]. Patients with diabetic foot ulcers whose blood glucose levels are poorly controlled exhibit higher mortality rates [28]. Our results indicate that male diabetic patients with MDRGNB-infected foot ulcers have poor glycemic control. Hence, male diabetic patients having MDRGNB-infected foot ulcers might have higher mortality than their female counterparts. Studies have reported 'male sex' as a significant risk factor for non-healing foot ulcers [29]. In a study by Basit et al. [30] hypertension was found to be significantly associated with poor glycemic control (OR = 1.65). Also, in a study by Minh et al. [31], the prevalence of hypertension was found to be significantly higher among men than women. Accordingly in our study, taking the absence of hypertension as a baseline (OR = 1.00), presence of hypertension with a presence of 'glycemic control at discharge' showed an OR of 0.375 while the same with an absence of 'glycemic control at discharge' showed an OR of 18.0. Hence, an absence of glycemic control with the presence of hypertension appears to be a strong indicator of MDRGNB-infections in male diabetic patients.

A surveillance program reported the frequency of ESBL-producing *K. pneumoniae *to be approximately 37% in Latin America vs. 7% in the United States [32]. Plasmid mediated *bla*_CTX-M-15 _among clinical isolates have also been reported earlier [33]. Only 2 of our 43 ESBL-producing strains were PCR-positive for *bla*_CTX-M-15_. This is in harmony with a study in which the authors detected only 3 strains out of 365 clinical isolates that were PCR-positive for *bla*_CTX-M-15 _[34]. However, this observation was contrary to many findings, which have reported a high prevalence of *bla*_CTX-M-15 _among clinical isolates [35]. ESBL-production in the remaining ESBL-positive strains might be due to ESBL-types other than CTX-Ms such as SHV/OXA/TEM/GES/BES/PER/TLA/IBC etc. as each of these have many variants. A high prevalence of ESBL-producers was observed in strains of *P. aeruginosa *(32.8%), which is generally considered to be a rare ESBL-producer. This result was in harmony with the reports from Peshawar, Pakistan (close to North India) and even Delhi (India) which is adjacent to Aligarh. The authors of these studies have reported 35.85% and 33.6% ESBL-producers among *P. aeruginosa *isolates, respectively [36,37]. However, since the sample sizes were not large, we prefer to conclude that the observations regarding high prevalence of ESBL-producers among *P. aeruginosa *strains made in these studies as well as ours might have been simply matters of coincidence. Class1 integrons are the most commonly found integron type in Gram-negative bacteria [38]. Accordingly, we detected 7 class1 integrons all of which were invariably PCR-positive for *int1 *and *sul1*. None of the *bla*_CTX-M-15 _markers were found to be located within the integrons. Hence, it is confirmed that the horizontal transfer of these markers was not integron-mediated but whole plasmid-mediated in our setting. Authors have earlier reported whole plasmid-mediated transfer of *bla*_CTX-M-15 _despite the presence of integron [39]. Transconjugation experiments were successful in case of both the *bla*_CTX-M-15 _positive *E. coli *strains in the present study. It is important to mention here that in medical literature, not all transfer experiments were successful with ESBL-producing *E. coli*. In a study, 19 of 24 strains did not transfer the ESBLs [40]. Similarly only 38% success was attained in transfer of markers by conjugation in another study [41]. The presence of strains co-resistant to aminoglycosides, quinolones and third generation cephalosporins as observed in our setting is a matter of due concern. Such strains if combined with the production of carbapenemase might result in therapeutic dead-ends [42,43].

This is the first study to report modeling of CTX-M-15 enzyme which is one of the most common ESBLs in India and its docking with cefotaxime. Moreover, there is no entry for CTX-M-15 in the Protein Data Bank to date. Residues specifically in the active site omega loop (position 161-179) of CTX-M enzymes play an important role in the substrate profile for cephalosporins [44]. But residues outside the omega loop might also help in fixing cefotaxime into the active site of the enzyme, such as Asn-132 [45]. Accordingly we found that the residues Asn132, Glu166, Pro167, Val172, Lys234 and Thr235 make important contacts with cefotaxime in the docked complex. The SDN (positions 130-132), and KTG (positions 234-236) sequences, were found to be conserved, which are typical structure of class A enzymes [46]. The sequences harbored the D240G mutation with respect to CTX-M-3, the distinguishing feature of CTX-M-15 enzymes. None of the mutations obtained in our CTX-M-15 sequences lied within the omega loop or other positions that are known to affect catalytic properties or substrate profile of these enzymes. Hence the MIC values for cefotaxime and ceftazidime antibiotics in these isolates were typical of CTX-M-15 producers (Table 2). The *bla*_CTX-M-15 _positive isolates displayed cefotaxime MICs up to 8 fold higher than ceftazidime MICs. The strain having a higher cefotaxime MIC displayed a higher negative binding energy as well (Table 1). It can be explained by the fact that a higher negative value of binding energy for the enzyme-antibiotic complex is an indicator of more stable and effective binding. A more stable and better fitting of the β-lactam antibiotic (cefotaxime) into the active site of the CTX-M-15 enzyme would ensure an easy hydrolysis of the drug. This is in coherence with one of our earlier observations in which we performed docking of the enzyme Sme1 with different carbapenems to compare their effectiveness against Sme1 producing bacteria [47]. It was observed that the imipenem-Sme1 complex was far more stable than the complex involving doripenem, the overall most effective carbapenem to date. This suggested an easier hydrolysis of imipenem by Sme-1 and a poor hydrolysis of doripenem.

## Conclusions

The following conclusions can be drawn from this study: **(*i*) **Male diabetic patients with MDRGNB-infected foot ulcers have poor glycemic control and hence they might have higher mortality rates compared to their female counterparts **(*ii*) **Plasmid-mediated conjugal transfer, albeit at a low frequency might be the possible mechanism of transfer of *bla*_CTX-M-15 _resistance marker in the present setting. **(*iii*) **Since the docking results proved that the amino acid residues Asn132, Glu166, Pro167, Val172, Lys234 and Thr235 of CTX-M-15 (enzyme) make important contacts with cefotaxime (drug) in the 'enzyme-drug complex', researchers are expected to duly utilize this information for designing more potent and versatile CTX-M-inhibitors.

## Competing interests

The authors declare that they have no competing interests.

## Authors' contributions

SS has performed all the experiments incorporated in this manuscript. AUK has designed the problem, approved the final draft of the manuscript and was a guide throughout this study.
